# Diverse *Burkholderia* Species Isolated from Soils in the Southern United States with No Evidence of *B*. *pseudomallei*

**DOI:** 10.1371/journal.pone.0143254

**Published:** 2015-11-23

**Authors:** Carina M. Hall, Joseph D. Busch, Kenzie Shippy, Christopher J. Allender, Mirjam Kaestli, Mark Mayo, Jason W. Sahl, James M. Schupp, Rebecca E. Colman, Paul Keim, Bart J. Currie, David M. Wagner

**Affiliations:** 1 Center for Microbial Genetics and Genomics, Northern Arizona University, 1395 S Knoles Drive, Flagstaff, AZ 86011, United States of America; 2 Menzies School of Health Research, Casuarina, NT, Australia; 3 Translational Genomics Research Institute, Flagstaff, AZ 86001, United States of America; Ghent University, BELGIUM

## Abstract

The global distribution of the soil-dwelling bacterium *Burkholderia pseudomallei*, causative agent of melioidosis, is poorly understood. We used established culturing methods developed for *B*. *pseudomallei* to isolate *Burkholderia* species from soil collected at 18 sampling sites in three states in the southern United States (Arizona (*n* = 4), Florida (*n* = 7), and Louisiana (*n* = 7)). Using multi-locus sequence typing (MLST) of seven genes, we identified 35 *Burkholderia* isolates from these soil samples. All species belonged to the *B*. *cepacia* complex (Bcc), including *B*. *cenocepacia*, *B*. *cepacia*, *B*. *contaminans*, *B*. *diffusa*, *B*. *metallica*, *B*. *seminalis*, *B*. *vietnamiensis* and two unnamed members of the Bcc. The MLST analysis provided a high level of resolution among and within these species. Despite previous clinical cases within the U.S. involving *B*. *pseudomallei* and its close phylogenetic relatives, we did not isolate any of these taxa. The Bcc contains a number of opportunistic pathogens that cause infections in cystic fibrosis patients. Interestingly, we found that *B*. *vietnamiensis* was present in soil from all three states, suggesting it may be a common component in southern U.S. soils. Most of the *Burkholderia* isolates collected in this study were from Florida (30/35; 86%), which may be due to the combination of relatively moist, sandy, and acidic soils found there compared to the other two states. We also investigated one MLST gene, *recA*, for its ability to identify species within *Burkholderia*. A 365bp fragment of *recA* recovered nearly the same species-level identification as MLST, thus demonstrating its cost effective utility when conducting environmental surveys for *Burkholderia*. Although we did not find *B*. *pseudomallei*, our findings document that other diverse *Burkholderia* species are present in soils in the southern United States.

## Introduction

The Gram-negative genus *Burkholderia* is composed primarily of diverse soil-dwelling bacteria that play a variety of ecological roles as saprophytes, nitrogen-fixing mutualists, and pathogens. The genus includes plant pathogens, such as B. gladioli and B. glumae, and two species that are highly pathogenic to humans and other animals (B. *pseudomallei* and B. *mallei*). *Burkholderia pseudomallei*, the causative agent of melioidosis, is endemic to Southeastern Asia and Australasia [1, 2]. However, melioidosis also is reported sporadically in other locations of the world, including the Americas, Africa, the Middle East, and various island communities [1]. As a result, the current known global distribution of B. *pseudomallei* is thought to be just “the tip of the iceberg” [3].

Seroreactivity to B. *pseudomallei* antigens has been observed in healthy U.S. individuals [4], possibly as a result of exposure to B. *pseudomallei* or genetic near neighbor species. Five naturally acquired human melioidosis cases [5, 6] and four patients infected with genetic near neighbors of B. *pseudomallei* (B. *oklahomensis* and B. *thailandensis*) [7, 8] have been described in the U.S. Despite the possible presence of B. *pseudomallei* and its close genetic near neighbors in North America, only B. *oklahomensis* and B. *thailandensis* have been cultured from environmental samples [5, 7, 8]. However, members of the more distantly related B. *cepacia* complex (Bcc), which contains numerous opportunistic human pathogens [9–11], are frequently isolated in North America.

The diverse taxa of the Bcc have received increased attention due to their importance to plants, agriculture, and human health. One species belonging to this group, B. *vietnamiensis*, has the ability to fix nitrogen, which allows it to form mutualistic relationships with rice plants [12]. A well-known strain of B. vietnamiensis (G4) is especially interesting due to its ability to degrade common organic pollutants. This strain was isolated from a wastewater treatment facility in Florida and is now used for bioremediation [13]. Other species within the Bcc (B. *ambifaria*, B. *cenocepacia*, B. *cepacia*) have been identified as significant pathogens to commercially valuable plants, such as onions and bananas [14, 15]. Several members of the Bcc, as well as the more distantly related B. *gladioli*, have been described as opportunistic pathogens, particularly in cystic fibrosis (CF) patients [16–18]. The top three Bcc species responsible for infections of American CF patients are B. *cenocepacia*, B. *multivorans*, and B. *vietnamiensis* [18–20].

Based on clinical cases in North America involving individuals infected with *B*. *pseudomallei* or its close genetic near neighbors [21], we suspected these species might be present in North American environments that are similar to those in melioidosis-endemic regions of Asia and Australia. In particular, *B*. *pseudomallei* and its near neighbor species are found in Australia and Asia in sandy, acidic, moist soils that are well-oxygenated, and protected from UV exposure [22, 23]. In endemic regions where melioidosis cases are common, *B*. *pseudomallei*, *B*. *thailandensis*, and *B*. *oklahomensis* can be readily isolated from soil and water samples using selective media developed for the isolation of *B*. *pseudomallei*, such as Ashdown’s agar [24]. A recent consortium outlined effective methods and media for conducting surveys of *B*. *pseudomallei* in the environment [25]. However, few surveys for *B*. *pseudomallei* and its near neighbors have been conducted outside of endemic areas [26, 27] despite the ongoing discovery of new species in this group [28]. To address this knowledge gap, we surveyed for *B*. *pseudomallei* and its genetic near neighbors in soils from three southern U.S. states using these well-established sampling and culturing methods.

## Methods

### Environmental sampling in the U.S

**Fig 1:**
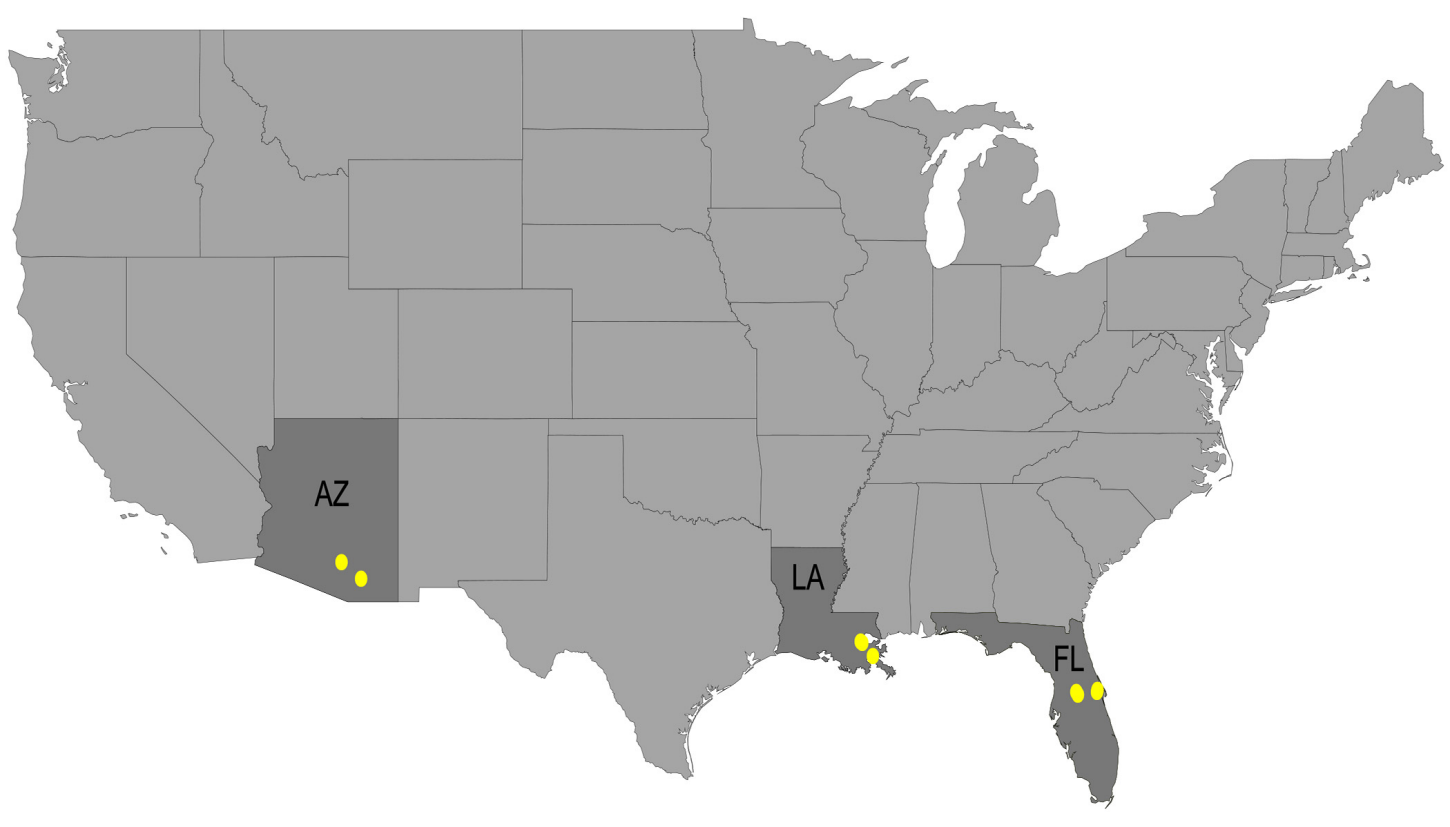
Soil sampling locations within the United States. Soil sampling was conducted in the states of Arizona (AZ), Louisiana (LA), and Florida (FL). Yellow circles indicate the specific locations. More location information is provided in Table 1 and S1 Fig.

From September-November 2012, we collected soil from three southern states (Arizona, Florida, and Louisiana) to survey for *B*. *pseudomallei* and its genetic near neighbors (Fig 1). Warm southern regions were selected because *B*. *pseudomallei* is largely endemic to tropical regions, such as Southeast Asia and northern Australia [1]. Due to their proximity to the hot and humid climate of the Gulf of Mexico, both Florida and Louisiana provide the closest approximation in the US to the environmental conditions where *B*. *pseudomallei* is endemic. Arizona was included due to a recent case of melioidosis described from the southern region of that state [5, 29]. All collection sites were chosen for their close vicinity to surface freshwater with road access, but we avoided agricultural fields and human dwellings (Table 1). No specific permissions were required for our sampling activities, because they occurred along the right-of-way of public roads and in municipal parks without access restrictions. Also, the field sampling did not involve endangered species or protected habitats. Soil collection procedures followed those employed by the Menzies School of Health and Research [22] and are expanded versions of those used by the wider *B*. *pseudomallei* research community [25]. Soil was collected at multiple sites per state (AZ = 4, FL = 7, LA = 7), which are displayed on maps in S1 Fig. At each site five sampling holes were dug along linear 40m transects (10m between holes) using hand spades, with two samples collected at different depths (10cm and 30cm) from each hole for a total of 180 soil samples. All field equipment was rinsed with water and decontaminated with 70% ethanol between each sampling hole. Soil samples were collected in 50mL sterile conical tubes and stored in the dark at ambient temperature until they were shipped (also at ambient temperatures) to Northern Arizona University.

**Table 1:** Soil sample collection sites for *Burkholderia* spp. isolation, with ten samples collected per site.

State ^a^	Site	# of *Burkholderia* isolates	Species ^b^	Collection Date	Dominant soil type at site	County	County average annual rainfall (inches) ^c^	Closest water source	Latitude (North)	Longitude (West)
AZ	1	0		9/24/2012	clay	Pinal	9.36	Cattle tank	32.56	-111.36
AZ	2	0		9/24/2012	clay	Pinal	9.36	Cattle tank	32.56	-111.36
AZ	3	1	B.v (1)	9/25/2012	sand	Pima	10.92	Coyote Springs	32.04	-110.56
AZ	4	1	B.a (1)	9/25/2012	sand	Pima	10.92	Coyote Springs	32.04	-110.56
FL	1	2	B.co (1); B.v (1)	11/6/2012	sand	Orange	51.12	St. Johns River	28.54	-80.94
FL	2	10	B.c (1); B.v (9)	11/6/2012	sand	Brevard	51.83	Fox Lake	28.59	-80.87
FL	3	0		11/6/2012	sand	Brevard	51.83	Salt Lake	28.64	-80.90
FL	4	1	B.c (1)	11/6/2012	sand	Brevard	51.83	Salt Lake	28.66	-80.91
FL	5	6	B.c (1); B.s (2); B.v (3)	11/7/2012	sand	Lake	51.63	Lake Minneola	28.56	-81.77
FL	6	3	B.ce (1); B.c (1); B.m (1)	11/7/2012	sand	Lake	51.63	Lake Minneola	28.56	-81.78
FL	7	8	B.c (1); B.d (1); B.v (6)	11/7/2012	sand	Lake	51.63	Trout Lake	28.45	-81.71
LA	1	0		10/10/2012	sand	Plaquemines	61.17	Mississippi River	29.66	-89.95
LA	2	1	B.d (1)	10/10/2012	sand	Plaquemines	61.17	Mississippi River	29.66	-89.95
LA	3	0		10/10/2012	clay	St. John the Baptist	62.25	Lake Maurepas	30.10	-90.44
LA	4	0		10/10/2012	clay	St. John the Baptist	62.25	Lake Maurepas	30.10	-90.44
LA	5	1	B.v (1)	10/10/2012	clay	St. John the Baptist	62.25	Lake Ponchartrain	30.11	-90.43
LA	6	1	B.v (1)	10/10/2012	sand	St. Charles	61.83	Lake Ponchartrain	30.06	-90.37
LA	7	0		10/10/2012	clay	St. Charles	61.83	Lake Ponchartrain	30.06	-90.37
**Total isolates**		**35**								

^a^ AZ = Arizona, FL = Florida, LA = Louisiana

^b^ B.a = *B*. *arboris*; B.ce *= B*. *cenocepacia*; B.c = *B*. *cepacia*; B.co = *B*. *contaminans*; B.d = *B*. *diffusa*; B.m = *B*. *metallica*; B.s = *B*. *seminalis*; B.v = *B*. *vietnamiensis*

^c^ Obtained from www.usa.com. Calculated from historical data of U.S weather stations from 1980 to 2010.

All culturing procedures were carried out at Northern Arizona University and followed methods previously described [25, 30]. It is important to note that these methods were developed specifically to isolate *B*. *pseudomallei* and not all *Burkholderia* species. Briefly, 20g of each collected soil sample was suspended in 20mL of sterile distilled water and incubated at 37°C while shaking (250 rpm) for 48 hours. Samples were allowed to settle for 1 hour and then 100μL of the water suspension was plated onto Ashdown’s agar plates. Also, 10mL of water suspension was inoculated into 10mL of Ashdown’s broth (containing 0.05 mg/mL colistin) [24]. The Ashdown’s broth was then shaken at 37°C for seven days. We plated 10μL of the top layer from the Ashdown’s broth suspension onto Ashdown’s agar plates (containing 4 mg/mL gentamycin) at Day 2 and Day 7 post broth inoculation. After 48 hours of incubation at 37°C, we sub-cultured single colonies from the Ashdown’s agar plates (up to 5 colonies per plate). We were specifically interested in isolating members of the *B*. *pseudomallei* group and, thus, selected colonies that displayed a morphology similar to *B*. *pseudomallei*: lavender to purple colonies, dry, slightly textured, with a raised dome or fried-egg morphology, and dimpled/wrinkled centers [31, 32]. Because we used these selection criteria and methods developed specifically for the isolation of *B*. *pseudomallei* and not all *Burkholderia* species, it is likely that we missed some *Burkholderia* species present in the samples that are not closely related to *B*. *pseudomallei* [33].

### Detection of *B*. *pseudomallei*

To quickly determine the presence of *B*. *pseudomallei* we screened DNA extractions using a real-time PCR assay that targets *orf2* in the type three secretion system 1 (TTS1) cluster of *B*. *pseudomallei*. This target is highly specific to *B*. *pseudomallei* and is considered the gold standard for PCR-based detection of *B*. *pseudomallei* [34]. Although the consensus guidelines suggest using the latex agglutination assay, we used molecular identification because it is more accurate. DNA was extracted from all sub-cultured colonies using a 5% Chelex®-100 heat soak method [35, 36]. All DNAs were screened using published conditions on ABI 7900 machines. We used DNA from a known positive control isolate of *B*. *pseudomallei* (K96243) and water was used for no-template controls (NTCs).

### Molecular identification of *Burkholderia* spp

To identify any potential *Burkholderia* spp. from the Ashdown’s medium, we sequenced a 365bp section of the recombinase A gene (*recA*) and, when necessary, the 16S *rRNA* gene. The RecA protein is essential for DNA recombination and repair and its nucleotide gene sequence exhibits mutations among *Burkholderia* species, making it valuable as a molecular target for species-level identification [37]. Since other soil-dwelling bacteria besides *Burkholderia* are known to grow on Ashdown’s media (including *Delftia*, *Pandoraea*, *Pseudomonas*, and *Ralstonia*) [31], we used *Burkholderia*-specific primers BUR 3 [38] and BUR5 [39] to amplify a 365bp region of the *recA* gene for the next screening of all sub-cultured isolates. The 20μL PCR contained final concentrations of the following reagents: 1 x buffer, 1.5mM MgCl_2_, 0.25mM dNTPs, 0.2μM of each primer, 1.0 U of Platinum^®^ *Taq* (Invitrogen, Grand Island, NY), 1.2M betaine, and 1μL of DNA template (diluted genomic DNA). A modified version of a previously described “slowdown PCR” designed for GC-rich template [40, 41] was used for cycle conditions (SEQSLOWD). The modifications made to the slowdown PCR were as follows: initial denature of 95°C for 5 minutes was excluded, extension time lengthened from 40 seconds to 3 minutes, starting annealing temperature reduced from 70°C to 65°C, ending annealing temperature reduced from 53°C to 52°C, starting annealing temperature reduced from 58°C to 55°C for the last 15 cycles, and final extension of 72°C for 10 minutes was added. The complete PCR cycle conditions for SEQSLOWD are listed in S1 Text. The 365bp PCR product (4μL) was visualized on a 1.5% agarose gel using a 100bp ladder (Invitrogen, Grand Island, NY) for reference to the target size and estimation of the dilution needed for cycle sequencing PCR (below).

The 16S *rRNA* gene was sequenced from isolates that yielded negative *recA* results to ensure that we did not exclude any *Burkholderia* spp. that may possess incompatible *recA* priming sites. We used modifications of universal 16S primers 27F.1G (forward, 5’-GAGRGTTTGATCMTGGCTCAG-3’) and 1391R (reverse, 5’-TGRACACACCGCCCGTC-3’) to amplify a ~1400bp region of the 16S *rRNA* gene [42]. The 20μL PCR contained final concentrations of the following reagents: 1 x buffer, 2.0 mM MgCl_2_, 0.2 mM dNTPs, 0.4μM of each primer, 1.6 U of Platinum^®^ *Taq* (Invitrogen, Grand Island, NY), and 1μL of DNA template (gDNA diluted 1/10). PCR cycle conditions were as follows: 5 min, 94°C; (30 sec, 94°C; 30 sec, 55°C; 75 sec, 72°C) x 35 cycles; 5 min, 72°C; held at 16°C. The 1400bp PCR product (4μL) was visualized on a 1.0% agarose gel using 1kb ladder (Invitrogen, Grand Island, NY) for reference to the target size and estimation of the required dilution for cycle sequencing PCR. Water was used for no-template controls (NTCs) and known *Burkholderia* DNA served as controls for all PCRs.

Both *recA* and 16S *rRNA* genes were sequenced using the Sanger method. To remove excess primers and dNTPs from the post-PCR product, 4μL of Exo-SAP-IT^®^ (USB Corporation, Cleveland, OH) was added to each reaction and incubated for 15 min at 37°C, followed by enzyme deactivation for 15 min at 80°C. PCR dilutions were made depending on the band intensity from the gel electrophoresis. Faint bands were diluted 1:2 in water whereas bright bands were diluted 1:5. The diluted PCR product was used as template for sequencing using the BigDye® Terminator v3.1 Cycle Sequencing Kit (Life Technologies, Grand Island, NY). The same primers used for the initial amplification of *recA* were used in two cycle sequencing reactions. An additional five internal primers (335F2, 5’-CTCCTACGGGAGGCAGCAG-3’; 926F, 5’-CTCCTACGGGAGGCAGCAG-3’; 926F, 5’-TTAAAACTCAAATGAATTGACGGGG-3’; 1053F, 5’-GTGCTGCATGGCTGTCGTCAG-3’; 515R, 5’-ATTACCGCGGCTGCTGGCAC-3’; 787R, 5’-ATTAGATACCCRNGTAGTCC-3’; 1391R, 5’-TGRACACACCGCCCGTC-3’) [42] were used to achieve full coverage of the 1400bp 16S *rRNA* gene in seven cycle sequencing reactions. Cycle sequencing conditions for *recA* and 16S *rRNA* genes were the same except for the primer starting concentrations (*recA*, 0.8μM; 16S *rRNA*, 3.2μM). The components for the cycle sequencing were 2μL 5x sequencing buffer, 1μL BigDye^®^ v3.1, 0.32μM of a single primer and 2μL of diluted PCR product producing a 10μL reaction. The cycle conditions for cycle sequencing consisted of 1 min, 96°C; (30 sec, 96°C; 10 sec, 50°C; 4 min, 60°C) x 30 cycles; held at 16°C. An EDTA/ethanol precipitation cleanup was performed on the products before they were sequenced on a 3130xl Sequencer (Applied Biosystems, Carlsbad, CA).

Once the *recA* and 16S rRNA fragments were sequenced they were assembled and edited by visual inspection with Sequencher 5.1 (Gene Codes, Ann Arbor, MI). Using NCBI _BLAST_ (http://blast.ncbi.nlm.nih.gov/Blast.cgi), all *recA* and 16S *rRNA* amplicons were identified to genus and any isolates that were not *Burkholderia* were excluded. The *Burkholderia recA* sequences were then aligned with other *Burkholderia recA* sequences from NCBI GenBank, including *B*. *xenovorans* as an outgroup taxon, as previously established by Cesarini et al., 2009 and Martina et al., 2013 [43, 44] (accession numbers reported in S2 Fig). A total of 65 sequences (35 sequences from this study and 30 external sequences) were aligned in Sequencher 5.1 using Clustal W Multiple Alignment. Aligned sequences were imported into MEGA version 5.2 to construct a maximum parsimony tree from the *recA* sequences with a bootstrapping method [45–47]. Only bootstrap values of ≥50% are reported on the consensus tree (S2 Fig).

### Multiple locus sequence type (MLST) analysis

Isolates identified as *Burkholderia* spp. were streaked from a single colony to form a lawn and then stored at -80°C in Luria Bertani (LB) broth with 20% glycerol. Culture was grown on LBA plates and incubated at 37°C for 24–48 hours. High molecular weight DNA was extracted using the Qiagen® DNeasy Blood and Tissue Kit (catalog no. 69504; Valencia, CA) in preparation for whole genome sequencing using Illumina HiSeq, MiSeq, or GAIIx (Illumina, Inc.; San Diego, CA) sequencing technology. Using approximately 2.7μg of gDNA, libraries were prepared for whole genome sequencing as previously described [48].

**Fig 2:**
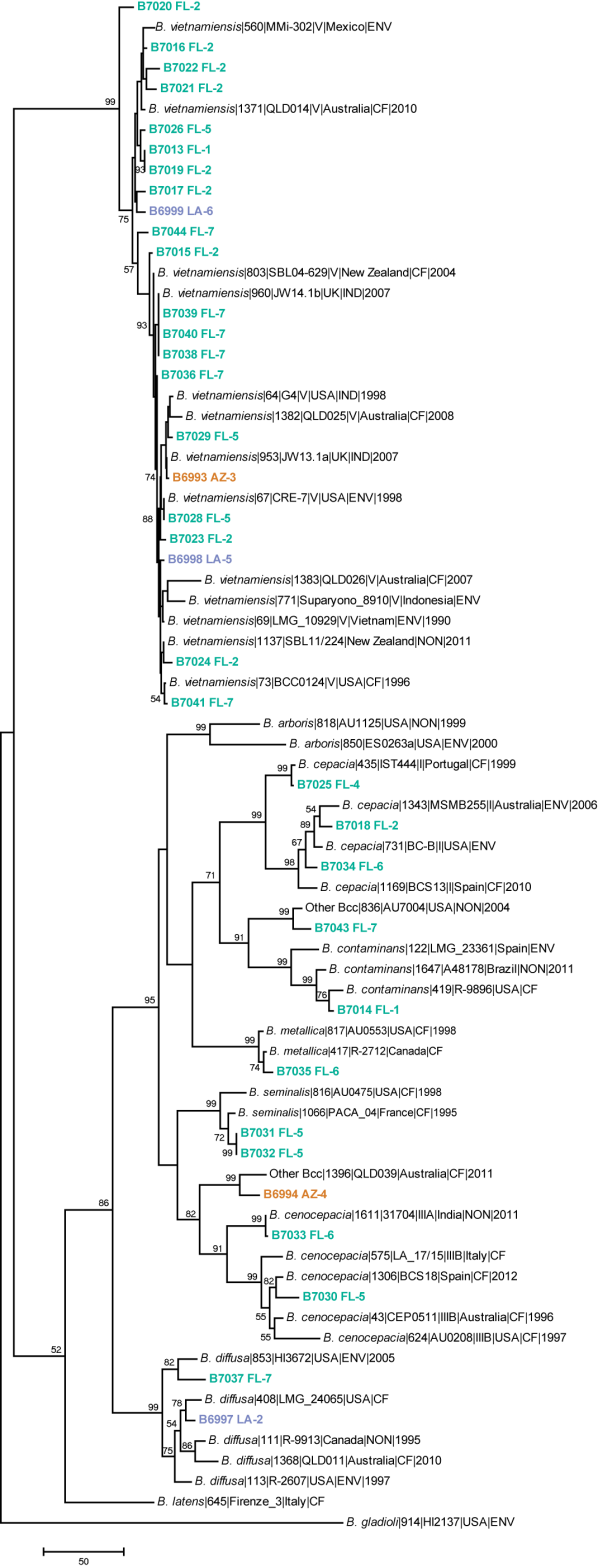
Maximum parsimony analysis of *Burkholderia* MLST gene sequences (2778bp) with 1,500 bootstrap replicates. All samples in bold font are isolates from this U.S. study and are labeled with a sample ID, collection state, and collection site. PubMLST sequences are labeled with species, PubMLST number, strain ID, genomovar (when available), collection country (when available), sample type (when available), and year (when available). Only bootstrap values ≥50% were reported. This tree was rooted with *B*. *gladioli*. The most parsimonious tree had a tree length of 1479 steps, a consistency index of 0.3960, and a retention index of 0.8650. Collection state: AZ = Arizona (orange text), FL = Florida (green), LA = Louisiana (purple). The tree is drawn to scale, with branch lengths calculated using the average pathway method and are in the units of the number of changes over the whole sequence. The analysis involved 75 nucleotide sequences.

Raw reads were assembled using SPAdes v3.5.0 [49] (data not shown). A multiple locus sequence type (MLST) system specific for the Bcc was used [50]. First, alleles were called by aligning assemblies against MLST alleles from the PubMLST website (see http://pubmlst.org/bcc/) with BLASTN [51]. For each case, the top BLAST hit for each allele was identified and the sequence type was reported for exact matches. For our 35 samples from this study, concatenated sequences of the seven MLST genes (*atpD*, *gltB*, *gyrB*, *recA*, *lepA*, *phaC*, and *trpB*) were generated by extracting the exact match from BLASTN alignments. Each sequence type (ST) was searched for in the PubMLST Bcc database (see http://pubmlst.org/bcc/) to determine its closest match. At least one representative was downloaded as an external reference in addition to the type strains for members of the Bcc and *B*. *gladioli* as a reference outside of the Bcc (40 reference sequences). All concatenated MLST sequences (*n* = 75) were aligned using MUSCLE [49] and a maximum parsimony analysis was performed using MEGA v6 with a bootstrapping method [45, 47, 52]. Only bootstrap values of ≥50% are reported on the consensus tree (Fig 2).

## Results/Discussion

### *Burkholderia* species identified

*Burkholderia pseudomallei* was not identified in any of our soil collections and, to date, this species has yet to be isolated from environmental samples within the United States. Despite the collection of both clinical and environmental isolates in the U.S. of *B*. *oklahomensis* [8] and *B*. *thailandensis* [7], we were unable to identify these species or any other close genetic near neighbors within the *B*. *pseudomallei* group (Fig 2). All 176 isolates sub-cultured from the Ashdown’s agar plates (AZ = 45, FL = 82, LA = 49) were negative for TTS1 but we identified 36 isolates that showed amplification using the *Burkholderia recA* assay. Of these 36 *recA* positive isolates, 35 were identified as *Burkholderia* spp., whereas the last isolate had a closest NCBI _BLAST_ identity with *Rubrivivax gelatinosus*. The 35 *Burkholderia* isolates were cultured from multiple locations in all states, including 2/4 (50%) sampling sites in AZ, 3/7 (43%) sampling sites in Louisiana, and 6/7 (86%) sampling sites in Florida (Table 2). All *Burkholderia* isolates, based upon a MLST maximum parsimony tree shown in Fig 2 (tree length: 1479 steps, consistency index: 0.3960, retention index: 0.8650), were members of the *B*. *cepacia* complex, including *B*. *cenocepacia* (*n* = 2), *B*. *cepacia* (*n* = 3), *B*. *contaminans* (*n* = 1), *B*. *diffusa* (*n* = 2), *B*. *metallica* (*n* = 1), *B*. *seminalis* (*n* = 2), and *B*. *vietnamiensis* (*n* = 22), and two other Bcc (Table 2 and Fig 2).

**Table 2:** All *Burkholderia* spp. isolated from U.S. soil during this study.

ID	Pub MLST #	State ^a^	Site	Sample	Depth (cm)	species ^b^	atpD	gltB	gyrB	recA	lepA	phaC	trpB	Sequence type	recA accession #
B6993	1658	AZ	3	1	30	*B*. *vietnamiensis*	27	20	15	22	12	11	17	200	KR011892
B6994	1662	AZ	4	2	10	Other BCC	106	274	302	339	68	295	205	1038*	KR011893
B7013	1863	FL	1	2	10	*B*. *vietnamiensis*	27	233	248	96	363	131	81	996*	KR011897
B7014	1864	FL	1	2	30	*B*. *contaminans*	89	422	113	71	39	54	70	1037*	KR011898
B7015	1870	FL	2	1	10	*B*. *vietnamiensis*	27	231	173	23	36	56	81	980*	KR011895
B7016	1872	FL	2	2	10	*B*. *vietnamiensis*	27	350	248	174	363	187	81	1033*	KR011899
B7017	1897	FL	2	2	30	*B*. *vietnamiensis*	27	231	248	23	163	131	81	984*	KR011900
B7018	1899	FL	2	3	10	*B*. *cepacia*	4	386	49	3	2	1	21	961*	KR011901
B7019	1900	FL	2	3	10	*B*. *vietnamiensis*	27	233	248	96	363	131	81	996*	KR011897
B7020	1901	FL	2	3	10	*B*. *vietnamiensis*	27	350	248	48	363	180	81	1031*	KR011902
B7021	1902	FL	2	3	30	*B*. *vietnamiensis*	27	350	223	174	36	131	188	1009*	KR011903
B7022	1904	FL	2	4	30	*B*. *vietnamiensis*	27	350	221	174	363	132	81	1005*	KR011903
B7023	1905	FL	2	5	10	*B*. *vietnamiensis*	27	19	16	22	12	187	17	969*	KR011904
B7024	1908	FL	2	5	30	*B*. *vietnamiensis*	27	19	107	23	12	11	268	974*	KR011895
B7025	1917	FL	4	2	10	*B*. *cepacia*	6	52	3	113	5	5	3	962*	KR011905
B7026	1922	FL	5	1	10	*B*. *vietnamiensis*	27	233	248	209	163	131	188	1000*	KR011906
B7028	1923	FL	5	2	10	*B*. *vietnamiensis*	27	19	15	22	36	11	17	63	KR011907
B7029	1924	FL	5	2	10	*B*. *vietnamiensis*	27	20	15	23	49	56	17	978*	KR011895
B7030	1925	FL	5	3	30	*B*. *cenocepacia*	67	175	326	49	94	8	122	1036*	KR011908
B7031	1926	FL	5	4	10	*B*. *seminalis*	165	161	328	144	156	123	45	1039*	KR011909
B7032	1927	FL	5	5	10	*B*. *seminalis*	165	161	328	144	156	123	45	1040*	KR011909
B7033	1928	FL	6	2	30	*B*. *cenocepacia*	15	11	487	14	11	6	147	964*	KR011910
B7034	1929	FL	6	3	10	*B*. *cepacia*	1	195	45	1	1	279	21	960*	KR011911
B7035	1930	FL	6	5	30	*B*. *metallica*	167	189	400	187	202	153	242	1041*	KR011912
B7036	1931	FL	7	1	10	*B*. *vietnamiensis*	27	231	15	22	12	56	17	979*	KR011904
B7037	1932	FL	7	2	10	*B*. *diffusa*	259	284	503	267	74	32	41	1042*	KR011913
B7038	1933	FL	7	2	10	*B*. *vietnamiensis*	27	231	202	22	49	56	17	600	KR011907
B7039	1934	FL	7	3	30	*B*. *vietnamiensis*	27	231	202	22	49	56	17	600	KR011904
B7040	1935	FL	7	4	10	*B*. *vietnamiensis*	27	231	202	22	49	56	17	600	KR011904
B7041	1936	FL	7	4	30	*B*. *vietnamiensis*	27	19	107	23	12	56	81	976*	KR011895
B7043	1937	FL	7	5	30	Other BCC	53	38	398	38	56	212	46	1034*	KR011914
B7044	1938	FL	7	5	30	*B*. *vietnamiensis*	27	233	248	23	12	180	81	987*	KR011895
B6997	1665	LA	2	3	30	*B*. *diffusa*	57	41	68	40	26	32	41	1035*	KR011894
B6998	1666	LA	5	5	30	*B*. *vietnamiensis*	27	19	16	23	12	56	17	380	KR011895
B6999	1695	LA	6	3	30	*B*. *vietnamiensis*	27	350	248	96	363	187	81	1032*	KR011896

^a^ AZ = Arizona, FL = Florida, LA = Louisiana

^b^ Species identified by placement within MLST phylogenetic tree (Fig 2).

* Represents a novel ST from this study

The state that yielded the most *Burkholderia* isolates was Florida with a total of 30 isolates, followed by Louisiana with three isolates, and Arizona with two isolates (S1 Fig). Since *B*. *pseudomallei* prefers moist soil [22] it was not surprising that Arizona, with the lowest average annual rainfall among these three states (Table 1), yielded the smallest number of isolates. All *Burkholderia* were cultured from sandy soils except for one strain that was isolated from a clay site in LA (Table 1). Both sampling depths yielded about equal numbers of Bcc isolates. One of the Florida sites in particular (site 2, Fox Lake, FL) provided a significant proportion (10/35, or 28%) of the total isolates. One explanation as to why more *Burkholderia* were isolated from Florida than Louisiana could be due to the pH of the soil. *Burkholderia* has been shown to have a higher tolerance for acidic soil than other bacteria found in soil [23, 53], and Florida has the greatest extent of low pH soil among the three states we sampled [54]. Although we did not collect pH data at our specific sampling locations, it is possible that the acidic soils in Florida could contribute to a higher abundance of *Burkholderia* compared to the other two states with higher soil pH. Of course, there will be local variations in soil pH within each of the states, which can influence the bacterial community on a fine scale.

A better understanding of U.S. soil microbial communities and their environmental conditions may provide important information about the presence and environmental preferences of opportunistic pathogens. A number of the Bcc species we recovered have been described as opportunistic pathogens, particularly in cystic fibrosis (CF) patients and other immunocompromised individuals [9, 11, 16–18]. In Florida, we isolated the two of the top three Bcc species responsible for the greatest number of infections in CF patients (B. *cenocepacia* and B. *vietnamiensis*) [10, 18–20]. Interestingly, we did not recover any B. *multivorans* from our soil samples despite the greater occurrence of this species in CF patients compared to all other members of the Bcc [18]. Based on the large number of CF clinical cases caused by these three species throughout the U.S., these *Burkholderia* spp. could be present in a wide range of soils throughout the U.S.

### *B*. *vietnamiensis* clade

Our study suggests that B. *vietnamiensis* may be a common component of the soil bacterial community in the southern U.S. We isolated B. *vietnamiensis* in all three states and it was the most common species in our survey (n = 22/35). As mentioned above, B. *vietnamiensis* is an opportunistic pathogen particularly in CF patients. However, it is also a beneficial species that can fix nitrogen in association with rice plants [12], and at least one strain (G4) is capable of degrading a common organic pollutant, trichloroethylene [13, 55]. The same sampling location that yielded B7020 (FL, site 2, sampling hole 3) also provided two other B. *vietnamiensis* strains with unique sequence types (STs) (B7019 FL-2 and B7021 FL-2), indicating that genetic diversity within a single soil collection hole can be quite high; a similar pattern has been found with B. *pseudomallei* [56]. Other members belonging to the Bcc could also be common components of the soil but we may have not sampled them due to our methods used for the preferential isolation of B. *pseudomallei*.

### Diversity within the *B*. *cepacia* complex

The MLST maximum parsimony tree (Fig 2) provided species-level resolution and demonstrated a substantial amount of diversity among our soil samples. Of the 35 *Burkholderia* isolates, six were assigned to currently defined STs whereas the other 29 represented 28 previously uncharacterized STs (Table 2). We obtained species-level identification for all isolates except two (B6994 and B7043). It appears that B6994 may represent a new lineage within the PubMLST database because its closest match was an undescribed member of the Bcc (QLD039) that shared the same allele at only three of seven MLST loci. The other exception, B7043, also had a closest match with an unknown member of the Bcc (AU7004) that shared alleles at five loci (Table 2 and Fig 2).

A short fragment from one of the MLST loci (*recA)* was capable of providing the same species identification as MLST loci in nearly all cases (33/35; see S2 Fig). Sequencing this smaller, relatively variable fragment of *recA* provides a more rapid and inexpensive tool for species identification of *Burkholderia* isolates compared to MLST. All SNPs within *recA* were synonymous mutations, as might be expected for a gene that is essential for DNA recombination and repair. Although MLST is a high resolution tool capable of recovering within-species diversity, *recA* by itself is able to provide adequate species-level resolution for molecular surveys of *Burkholderia* from environmental and clinical samples.

The isolates found in this study do not represent the overall community of *Burkholderia* species found in U.S. soils for two main reasons. First, we utilized a selective medium designed to isolate *B*. *pseudomallei* and it is likely that not all members of the Bcc grow on this medium. Second, the primary goal of this study was to attempt to isolate *B*. *pseudomallei* and, as a result, we preferentially selected colony morphologies similar to *B*. *pseudomallei*. During this selection process other members of the Bcc with less similar colony morphologies to *B*. *pseudomallei* could have been missed.

## Conclusions

All the *Burkholderia* species isolated from this study (with the exception of B. *metallica*) have been described from multiple continents and have world-wide distributions (see http://pubmlst.org/bcc/) [51]. We expected to find more *Burkholderia* in Florida and Louisiana than in Arizona since both states receive more than five times the amount of annual rainfall than Arizona (Table 1). A surprising result was that Florida yielded ten times as many *Burkholderia* isolates as Louisiana, as well as greater species diversity, although sampling sizes were low. One possible explanation to the dissimilarity in the number of *Burkholderia* isolates between Florida and Louisiana may be primarily due to soil type. Most soils sampled in Louisiana were of a heavy clay composition and probably had a neutral pH, whereas the Florida soils were predominantly sandy and more likely to be acidic. Clay-based soils are more likely to result in anaerobic conditions that are not ideal for the growth of *Burkholderia*, whereas an increased fraction of sand may increase the available oxygen and favor the survival of *Burkholderia*. Some soil dwelling bacteria have difficulty inhabiting soil with a lower pH whereas *Burkholderia* can tolerate a wide range of soil pH [23]. This provides *Burkholderia* with an advantage to survive in soils where other soil bacteria cannot and may be a contributor to why the majority *Burkholderia* isolates in this study were from Florida. Due to the lack of melioidosis cases in the U.S., we did not expect *B*. *pseudomallei* to be prevalent in our samples, especially considering our relatively small sample sizes. However, if *B*. *pseudomallei* is indeed present in isolated regions in the U.S., we hypothesize that Florida is the most likely of these three southern states to contain *B*. *pseudomallei*, based upon rainfall, soil type, and the results from this study.

## Supporting Information

S1 Fig Maps of soil sampling sites in the southern United States. Specific locations at which *Burkholderia* species were recovered are shown as red markers. Images generated in ArcMap 10.2 [57].
(DOCX)

S2 Fig Maximum parsimony analysis of *Burkholderia recA* gene sequences with 1,500 bootstrap replicates. All samples in bold font are isolates from this U.S. study and are labeled with a sample ID, collection state, collection site, and accession number. GenBank sequences are labeled with species, collection location (when available), sample type (when available), accession number, and strain ID. Only bootstrap values ≥50% were reported. This tree was rooted with *B*. *xenovorans*. The most parsimonious tree had a tree length of 217 steps, a consistency index of 0.4874, and a retention index of 0.8832. Collection state: AZ = Arizona (orange text), FL = Florida (green), LA = Louisiana (purple). Sample type: Cl = clinical, En = environmental. The tree is drawn to scale, with branch lengths calculated using the average pathway method and are in the units of the number of changes over the whole sequence. The analysis involved 65 nucleotide sequences.
(TIF)

S1 Text SEQSLOWD cycle conditions used for *recA* PCR. Modified version of previously described “slowdown PCR” designed for GC-rich template [40, 41].
(DOCX)
