# Supplementary material for: Diverse Burkholderia Species Isolated from Soils in the Southern United States with No Evidence of B. pseudomallei
Source: PLoS One. 2015 Nov 23;10(11):e0143254. doi: 10.1371/journal.pone.0143254 (PMC4658082; doi:10.1371/journal.pone.0143254)
Supplement: S1 Fig — Specific locations at which Burkholderia species were recovered are shown as red markers. Images generated in ArcMap 10.2 [57]. (DOCX) [file pone.0143254.s001.docx]

a) Four sites near Tucson, Arizona


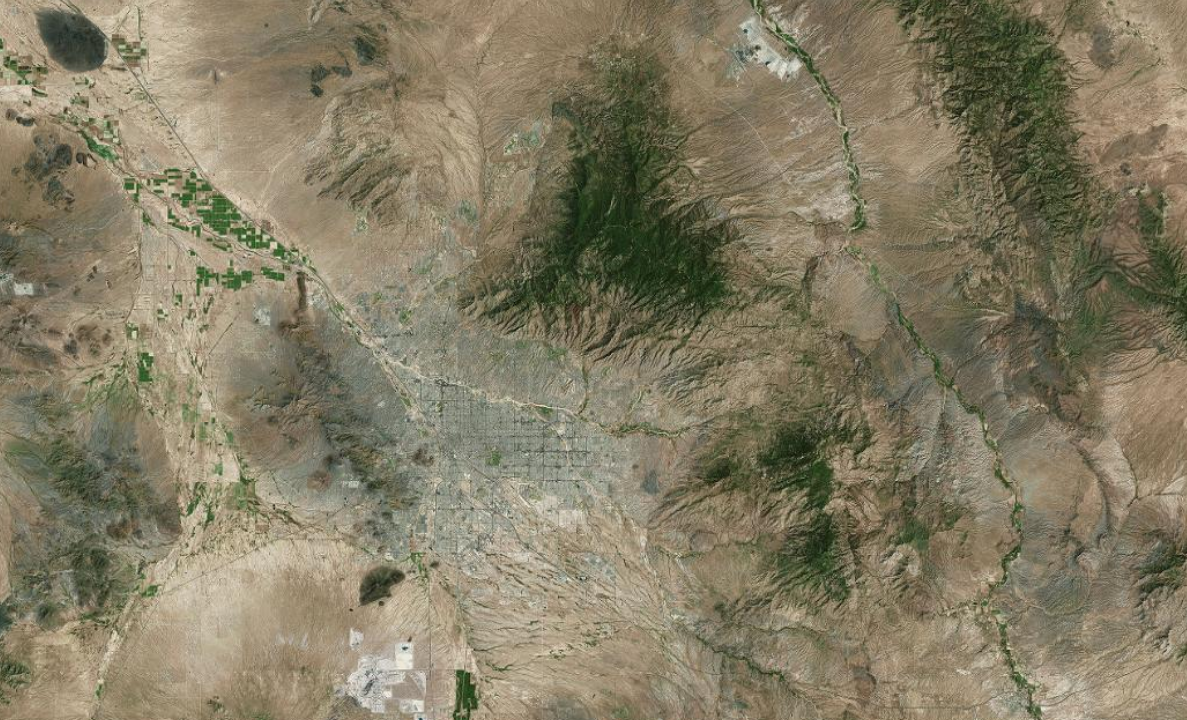


**AZ-1 and AZ-2:**

**No *Burkholderia* recovered**

**AZ-3:**

***B. vietnamiensis***

**AZ-4**

***B. arboris***

**T U C S O N**

**N**

b) Seven sites near New Orleans, Louisiana
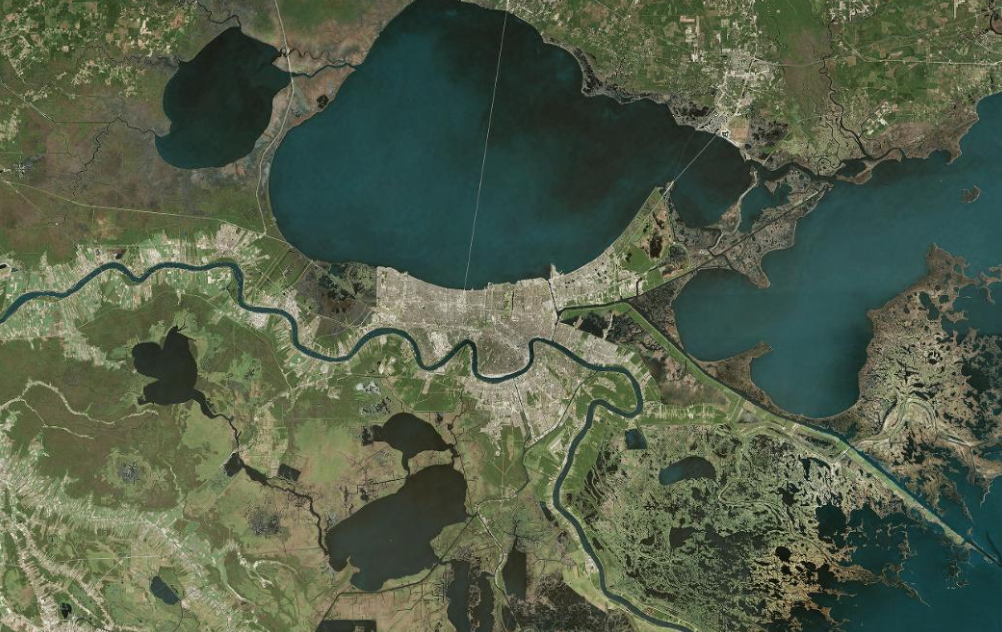


*Mississippi River*

**LA-2:**

***B. diffusa***

**LA-1:**

**No *Burkholderia* recovered**

**LA-5:**

***B. vietnamiensis***

**LA-3 and LA-4:**

**No *Burkholderia* recovered**

**LA-7:**

**No *Burkholderia* recovered**

**LA-6:**

***B. vietnamiensis***

**N E W O R L E A N S**

*Lake Pontchartrain*

**N**

c) Seven sites near Orlando, Florida


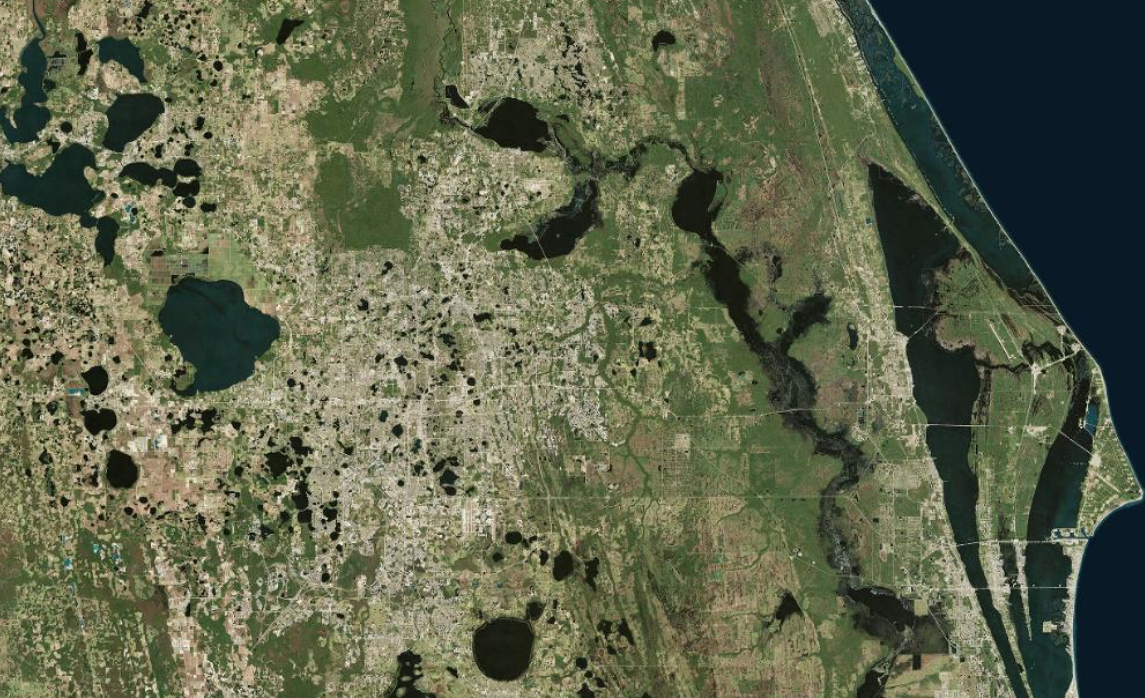


*Atlantic Ocean*

**N**

**O R L A N D O**

**FL-1:**

***B. contaminans***

***B. vietnamiensis***

**FL-4:**

***B. cepacia***

**FL-3:**

**No *Burkholderia* recovered**

**FL-2:**

***B. cepacia***

***B. vietnamiensis***

**FL-6:**

***B. cepacia***

***B. cenocepacia***

***B. metallica***

**FL-7:**

***B. cepacia***

***B. diffusa***

***B. vietnamiensis***

**FL-5:**

***B. cepacia***

***B. seminalis***

***B. vietnamiensis***
