# Supplementary figures and images for: Diverse Burkholderia Species Isolated from Soils in the Southern United States with No Evidence of B. pseudomallei
Source: PLoS One. 2015 Nov 23;10(11):e0143254. doi: 10.1371/journal.pone.0143254 (PMC4658082; doi:10.1371/journal.pone.0143254)

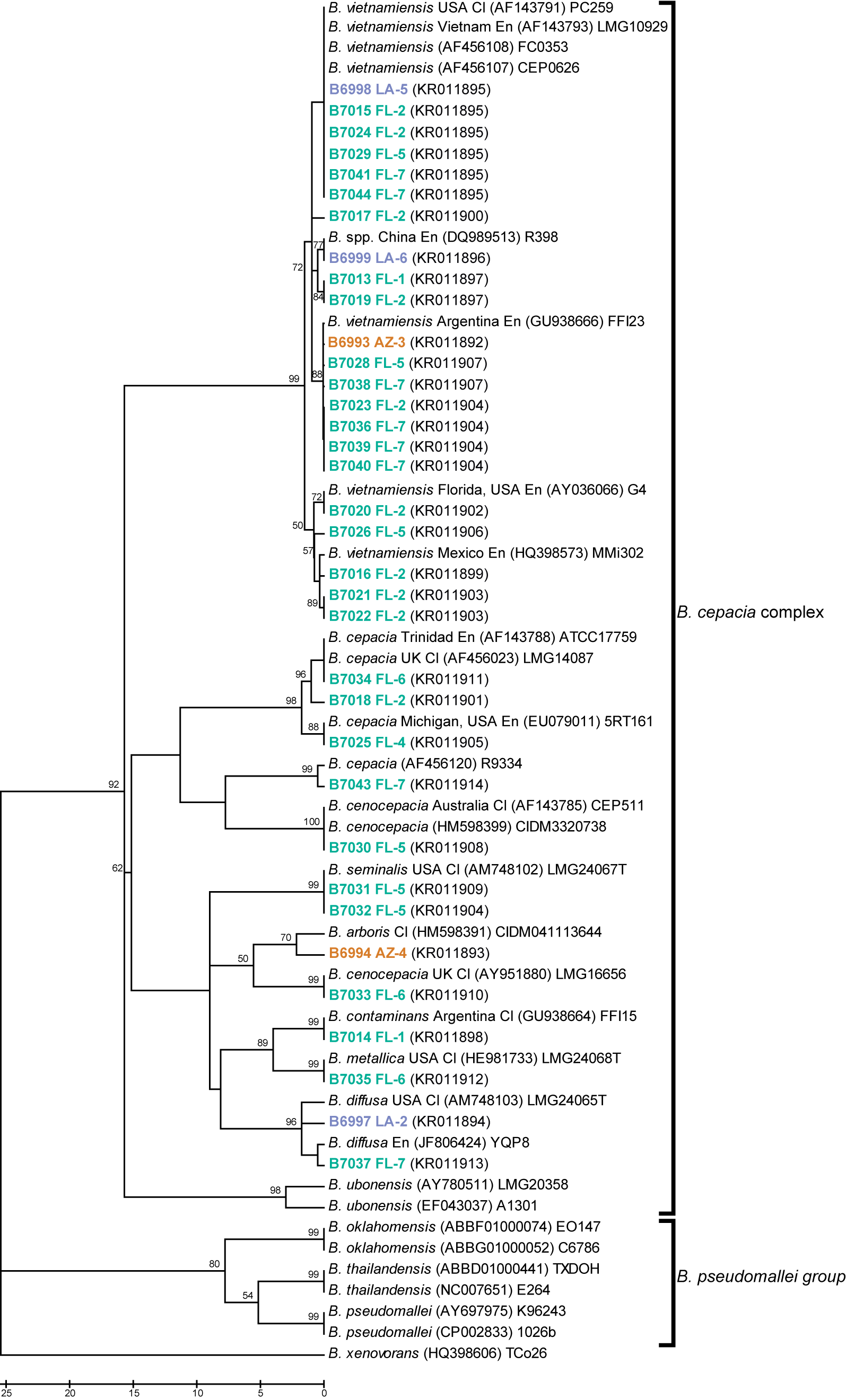

Supplement: S2 Fig — All samples in bold font are isolates from this U.S. study and are labeled with a sample ID, collection state, collection site, and accession number. GenBank sequences are labeled with species, collection location (when available), sample type (when available), accession number, and strain ID. Only bootstrap values ≥50% were reported. This tree was rooted with B. xenovorans. The most parsimonious tree had a tree length of 217 steps, a consistency index of 0.4874, and a retention index of 0.8832. Collection state: AZ = Arizona (orange text), FL = Florida (green), LA = Louisiana (purple). Sample type: Cl = clinical, En = environmental. The tree is drawn to scale, with branch lengths calculated using the average pathway method and are in the units of the number of changes over the whole sequence. The analysis involved 65 nucleotide sequences. (TIF) [file pone.0143254.s002.tif]
