# Supplementary material for: Diverse Burkholderia Species Isolated from Soils in the Southern United States with No Evidence of B. pseudomallei
Source: PLoS One. 2015 Nov 23;10(11):e0143254. doi: 10.1371/journal.pone.0143254 (PMC4658082; doi:10.1371/journal.pone.0143254)
Supplement: S1 Text — Modified version of previously described “slowdown PCR” designed for GC-rich template [40, 41]. (DOCX) [file pone.0143254.s003.docx]

Steps 1-7: ramp +2.5ºC/sec; 95ºC for 30 s; ramp -1.5ºC/sec; 65ºC for 30 s; ramp +2.5ºC/sec; 72ºC for 3 min (3x)

Steps 8-14: ramp +2.5ºC/sec; 95ºC for 30 s; ramp -1.5ºC/sec; 64ºC for 30 s; ramp +2.5ºC/sec; 72ºC for 3 min (3x)

Steps 15-21: ramp +2.5ºC/sec; 95ºC for 30 s; ramp -1.5ºC/sec; 63ºC for 30 s; ramp +2.5ºC/sec; 72ºC for 3 min (3x)

Steps 22-28: ramp +2.5ºC/sec; 95ºC for 30 s; ramp -1.5ºC/sec; 62ºC for 30 s; ramp +2.5ºC/sec; 72ºC for 3 min (3x)

Steps 29-35: ramp +2.5ºC/sec; 95ºC for 30 s; ramp -1.5ºC/sec; 61ºC for 30 s; ramp +2.5ºC/sec; 72ºC for 3 min (3x)

Steps 36-42: ramp +2.5ºC/sec; 95ºC for 30 s; ramp -1.5ºC/sec; 60ºC for 30 s; ramp +2.5ºC/sec; 72ºC for 3 min (3x)

Steps 43-49: ramp +2.5ºC/sec; 95ºC for 30 s; ramp -1.5ºC/sec; 59ºC for 30 s; ramp +2.5ºC/sec; 72ºC for 3 min (3x)

Steps 50-56: ramp +2.5ºC/sec; 95ºC for 30 s; ramp -1.5ºC/sec; 58ºC for 30 s; ramp +2.5ºC/sec; 72ºC for 3 min (3x)

Steps 57-63: ramp +2.5ºC/sec; 95ºC for 30 s; ramp -1.5ºC/sec; 57ºC for 30 s; ramp +2.5ºC/sec; 72ºC for 3 min (3x)

Steps 64-70: ramp +2.5ºC/sec; 95ºC for 30 s; ramp -1.5ºC/sec; 56ºC for 30 s; ramp +2.5ºC/sec; 72ºC for 3 min (3x)

Steps 71-77: ramp +2.5ºC/sec; 95ºC for 30 s; ramp -1.5ºC/sec; 55ºC for 30 s; ramp +2.5ºC/sec; 72ºC for 3 min (3x)

Steps 78-84: ramp +2.5ºC/sec; 95ºC for 30 s; ramp -1.5ºC/sec; 54ºC for 30 s; ramp +2.5ºC/sec; 72ºC for 3 min (3x)

Steps 85-91: ramp +2.5ºC/sec; 95ºC for 30 s; ramp -1.5ºC/sec; 53ºC for 30 s; ramp +2.5ºC/sec; 72ºC for 3 min (3x)

Steps 92-98: ramp +2.5ºC/sec; 95ºC for 30 s; ramp -1.5ºC/sec; 52ºC for 30 s; ramp +2.5ºC/sec; 72ºC for 3 min (3x)

Steps 99-105: ramp +2.5ºC/sec; 95ºC for 30 s; ramp -1.5ºC/sec; 55ºC for 30 s; ramp +2.5ºC/sec; 72ºC for 3 min (15 additional cycles)

Step 106: 72ºC for 10 min

Step 107: 4ºC for ever
